# Mitochondrial dysfunction causes Ca^2+^ overload and ECM degradation–mediated muscle damage in *C. elegans*

**DOI:** 10.1096/fj.201802298R

**Published:** 2019-06-04

**Authors:** Surabhi Sudevan, Mai Takiura, Yukihiko Kubota, Nahoko Higashitani, Michael Cooke, Rebecca A. Ellwood, Timothy Etheridge, Nathaniel J. Szewczyk, Atsushi Higashitani

**Affiliations:** * Graduate School of Life Sciences, Tohoku University, Sendai, Japan;; † College of Life and Environmental Science, University of Exeter, Exeter, United Kingdom;; ‡ Medical Research Council (MRC) and Arthritis Research United Kingdom (ARUK) Centre of Musculoskeletal Ageing Research and National Institute for Health Research, Nottingham Biomedical Research Centre, University of Nottingham, Nottingham, United Kingdom

**Keywords:** Antimycin A, collagen, Furin, DMD, MMP

## Abstract

Mitochondrial dysfunction impairs muscle health and causes subsequent muscle wasting. This study explores the role of mitochondrial dysfunction as an intramuscular signal for the extracellular matrix (ECM)–based proteolysis and, consequentially, muscle cell dystrophy. We found that inhibition of the mitochondrial electron transport chain causes paralysis as well as muscle structural damage in the nematode *Caenorhabditis elegans*. This was associated with a significant decline in collagen content. Both paralysis and muscle damage could be rescued with collagen IV overexpression, matrix metalloproteinase (MMP), and Furin inhibitors in Antimycin A–treated animal as well as in the *C. elegans* Duchenne muscular dystrophy model. Additionally, muscle cytosolic calcium increased in the Antimycin A–treated worms, and its down-regulation rescued the muscle damage, suggesting that calcium overload acts as one of the early triggers and activates Furin and MMPs for collagen degradation. In conclusion, we have established ECM degradation as an important pathway of muscle damage.—Sudevan, S., Takiura, M., Kubota, Y., Higashitani, N., Cooke, M., Ellwood, R. A., Etheridge, T., Szewczyk, N. J., Higashitani, A. Mitochondrial dysfunction causes Ca^2+^ overload and ECM degradation–mediated muscle damage in *C. elegans*.

Mitochondrial dysfunction is a frequent characteristic of several diseases that affect the muscle cell, including muscle wasting, muscular dystrophy, aging, and disuse (1–3). Irrespective of mitochondrial dysfunction, there are several well-known pathways for myofibril protein degradation: lysosome-mediated degradation, autophagy, caspase-mediated degradation, and calpain-mediated degradation (4, 5). Although these muscle-regulatory degradation systems are activated and performed intracellularly, recent studies have reported the importance of the extracellular matrix (ECM) in maintaining muscle cell activity and function (6, 7). The ECM is the physical support and biochemical environment that surrounds a cell and is composed of several components, including collagen, which is the most abundant ECM protein (8). Collagen VI connects skeletal muscle with the basement membrane, and hence is important in relaying bilateral biochemical messages from inside cells to the ECM and *vice versa* (9). From previous studies, it is clear that mitochondrial dysfunction is related to muscle atrophy (10), and ECM stability is necessary for maintaining muscle health; however, any putative molecular mechanisms linking the mitochondria, ECM, and muscle are unclear.

The present study addresses the gap in information about the pathway from mitochondrial dysfunction to muscle atrophy using *Caenorhabditis elegans*, an experimental model organism. *C. elegans* is a powerful tool to study muscle decline and health because there are exactly 95 body wall muscle cells, and the fate of each muscle cell from its birth to death can be determined easily. In addition, in our own heat stroke model system of *C. elegans*, we found that calcium up-regulation leads to disorder of body wall muscle cells (11). Through the present study, we report for the first time that mitochondrial dysfunction acts as an intramuscular signal that, *via* excessive calcium release, activates ECM-degrading enzymes to reduce ECM content and, subsequently, target muscle structural and functional decline.

## MATERIALS AND METHODS

### *C. elegans* strains and culturing techniques

All strains were cultured on Nematode growth medium (NGM) plates with OP50 as a food source at 20°C unless otherwise specified. In the case of temperature-sensitive (TS) mutants, the worms were grown at a permissive temperature (15°C) and transferred to a restrictive temperature (25°C) for the experiment at the specified time period. Standard protocols were followed for the maintenance of all *C. elegans* strains (12). For RNA interference (RNAi) experiments, adult worms were transferred to plates containing isopropyl β-D-1-thiogalactopyranoside and bacteria expressing double-stranded RNA for *unc-68* or *emb-9*. The nematode strains used in this study were Bristol strain N2 (WT, wild type), GG34 [*emb-9*(*g34*) III], HBR4 {*goeIs3* [myo*-3p::GCamP3.35::unc54 3′utr + unc-119*(*+*)]}, NF3620 {*unc-119*(*ed3*) III; *tkTi1* [*emb-9p::emb-9::mCherry*+*Cb-unc-119*(*+*)]} I], TR2171 [*unc-68*(*r1162*) V], VC48 [*kpc-1*(*gk8*) I], and LS587 [*dys-1*(*cx18*) I; *hlh-1*(*cc561*) II].

### Reagents

Antimycin A (ALX-380-075; Enzo Life Sciences, Farmingdale, NY, USA) dissolved in 100% ethanol was used for inhibition of mitochondrial function. Furin inhibitor I (150113-99-8; Cayman Chemicals, Ann Arbor, MI, USA) and actinonin (13434-13-4; Cayman Chemicals) were dissolved in 100% DMSO and used to inhibit Furin and matrix metalloproteinases (MMPs) at the final concentration of 1 and 10 µM, respectively. Rhodamine phalloidin (219920-04-4) was dissolved in 100% methanol and was used for staining and visualizing muscle fibers. Rotenone (R8875; MilliporeSigma, Burlington, MA, USA) dissolved in 100% DMSO was used for inhibition of complex I of the electron transport chain.

### Muscle paralysis assay and morphology

Adult animals of N2 or other strains of *C. elegans* (*n* = 20 worms/condition) were treated with different concentrations (0, 2, 4, and 10 µM) of Antimycin A. After 36 h of treatment at 20°C, the animals were gently touched using a platinum wire pick to determine whether they were motile for the paralysis assay. For visualizing the muscle, after treatment with the drugs, the animals (*n* = 20) were washed thrice with M9, fixed using 1% paraformaldehyde for 10 min, and stored at 4°C. They were then washed twice with M9 and permeabilized using 100% acetone for 1 min. This was followed by 2 more washes with M9 and the addition of rhodamine phalloidin (12 U/ml). The worms were incubated in the dye in dark conditions for 2 h and then mounted on glass slides. Confocal microscopy (FluoView Olympus FV10i; Olympus, Tokyo, Japan) was used to visualize and image the myofibrils. In the case of Furin (1 µM) or MMP (10 µM) inhibition, the inhibitors were added at the same time as Antimycin A.

### Protein extraction and Western blot analysis

Following treatment with Antimycin A, animals expressing EMB-9::mCherry (*n* = 300) were collected in 1.5-ml microfuge tubes. After 2 washes with M9 buffer, animals were immediately frozen in liquid N_2_. This was followed by thawing and addition of 100 µl of 1-time SDS dye. Next, the tubes were placed in a liquid bath sonicator (Bioruptor; Cosmo Bio, Tokyo, Japan) on 5-s on and off cycles for 10 min, followed by incubation at 95°C for 10 min. Samples were then run on an 8% SDS gel, after which they were transferred onto a PVDF membrane (88518; Thermo Fisher Scientific, Waltham, MA, USA) and blocked in 5% skimmed milk for 1 h. Next, the membrane was incubated overnight at 4°C in primary antibody (anti-mCherry, ab125096, anti-α-tubulin, ab7750; Abcam, Cambridge, MA, USA) prepared in 2.5% milk. This was followed by 1 wash with 1-time PBS with Tween 20 for 5 min, and the membrane was transferred to secondary antibody (sheep anti-mouse; GE Healthcare, Waukesha, WI, USA; NA93IV, po448, goat anti-rabbit, Agilent Technologies, Santa Clara, CA, USA) incubated at room temperature for 3 h. The membrane was washed thrice with 1-time PBS with Tween 20, and the bands were then visualized using a chemifluorescence kit (Western Lightning Plus-ECL; Takara, Kyoto, Japan).

### Calcium assay method

HBR4 {*goeIs3* [myo*-3p::GCamP3.35::unc54 3′utr + unc-119*(*+*)]} was used to assay calcium concentration in the body wall muscles of *C. elegans* after 36 h treatment with Antimycin A. Analysis was done using a BX51 fluorescent microscope (Olympus) with a DC73 charge-coupled device camera (Olympus) and an FV10i confocal laser scanning microscope (Olympus). Green fluorescent protein intensity was measured using ImageJ (National Institutes of Health, Bethesda, MD, USA) software.

### ATP assay

Adult synchronized animals of wild-type (WT) *C. elegans* were treated with different concentrations (0, 2, 4, and 10 µM) of Antimycin A for 24 h, after which the endogenous ATP levels were measured by using an ATP Determination Kit (Molecular Probes, Eugene, OR, USA) as previously described by Momma *et al*. (11).

### Mass spectrometry

The synchronized adult animals (∼700 worms each treated with 0, 4, and 10 µM Antimycin A for 24 h) were washed with M9 buffer, and their total protein was extracted with 8.4 M urea solution and sonication (UR-20P; Tomy Seiko, Tokyo, Japan). The crude extracts were denatured, reduced, and digested with trypsin overnight at 37°C (13). The digested peptides were labeled with Applied Biosystems iTraq Reagents according to the manufacturer’s instructions (Thermo Fisher Scientific). Each peptide was quantitatively analyzed using an LTQ Orbitrap Velos with an electron-transfer dissociation mass spectrometer (Thermo Fisher Scientific) equipped with a PAL HTC‐xt autosampler (PAL System; American Medical Response, Greenwood Village, CO, USA) and a nano-Advance UHPLC system (Bruker, Billerica, MA, USA) as previously described by Pancha *et al*. (14).

### Oxygen-consumption rate assay

All control and Antimycin A– or rotenone-treated animals were prepared as described above for analysis of 36-h postadulthood animals. Seahorse XF^e^24 flux assay kits (Agilent Technologies) were hydrated using 1 ml of Seahorse XF calibration solution in each well and incubated overnight at 37°C in a humidified non-CO_2_ incubator. The Seahorse 24-well plate reader was also allowed to equilibrate overnight with the temperature set to 18°C. Plate template was set up using assay wizard to provide a random distribution of conditions across the plate. When preparing the animals, samples were washed (3 times, 3 × 10 min) to rinse excess bacteria and clear the gut of *E. coli*. Approximately 20 animals were counted/well (and precise worm count recorded) with 5 replicates/condition and a well volume of 525 µl M9 buffer. Basal respiration measurements were taken for 4 cycles for calculation of oxygen-consumption rate (OCR). Each measurement cycle consisted of a 2-min mix step, a 3-min wait step, and a 3-min measuring period. All conditions were analyzed over 2 Seahorse experiments for a total of 10 replicates of 20 worms/condition. Absolute OCR measurements were normalized/worm.

### Statistical analysis

RStudio software (*https://www.rstudio.com/*) was used to determine statistical significance. Statistical analysis was performed using 1-way ANOVA followed by Tukey’s *post hoc* test. The minimum *P* value for significance was 0.05. Similar letters in any 2 groups indicate no significance, and different letters in any 2 groups represent significant difference between the 2 groups.

## RESULTS

### Mitochondrial dysfunction mediated by Antimycin A results in muscle damage in *C. elegans*

**Figure 1:**
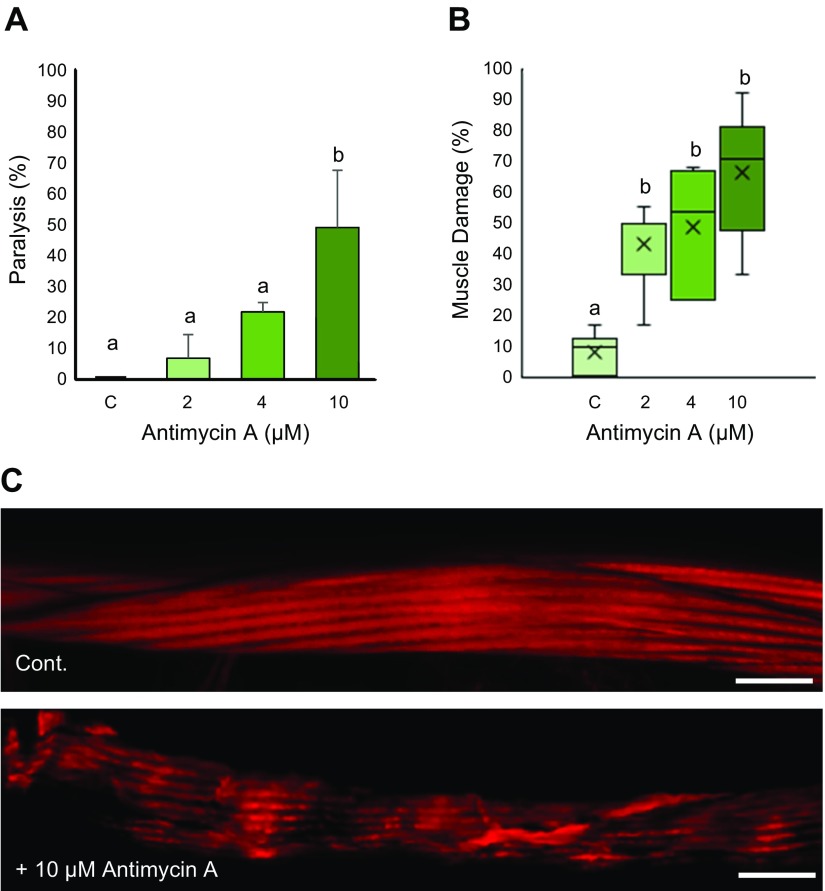
Mitochondrial dysfunction mediated by Antimycin A results in paralysis and muscle cell damage in *C. elegans*. Age-synchronized WT animals were grown to young adulthood at 20°C. *A*) Adult animals were then treated with 0 (C, control), 2, 4, and 10 µM Antimycin A for 36 h, after which the animals were analyzed with a platinum wire pick for movement defects/paralysis. Values indicate the percentage of animals paralyzed in each group. Twenty animals were analyzed for each experiment with 3 independent repetitions (*n* = 60 animals). A significant increase in paralysis was observed in 10 µM concentration. *B*) Muscle structure was visualized using rhodamine phalloidin after 36 h treatment with Antimycin A. Values indicate the average percentage of damaged muscle/animal. A significant increase in damaged muscle cells was observed in 2, 4, and 10 µM Antimycin A. Number of muscle cells/condition (*n* = 63, 54, 74, and 85 from left to right). *C*) Representative images of myofilament alignment in normal and Antimycin A–damaged muscle cells. The damaged muscle displays wavy myofilaments and large gaps in between compared with normal muscle cells. Cont., control. Scale bars, 10 µM. Letters (*a, b*) on top of bars indicate statistical significance; X, median value.

Treatment with a mitochondrial electron transport chain inhibitor Antimycin A resulted in worm paralysis as well as muscle damage in WT worms (**Fig. 1*A, B***). Almost 50% of adult worms were paralyzed after treatment with 10 µM Antimycin A, whereas 2 and 4 µM treatment exhibit ∼7 and 20% paralysis, indicating muscular functional decline as a direct consequence of mitochondrial dysfunction. Analysis of muscle structure using rhodamine phalloidin further reveals abnormal muscle structure, which resembles the dystrophy observed in *dys-1; hlh-1* mutant animals (15). Varying degrees of muscle damage were observed in these worms. The most common abnormal structural phenotype observed in these cases is that of wavy actin filaments compared with the straight parallel alignment observed in healthy muscle cells. In some cases, however, we also observe severely damaged muscle cells with broken myofilaments (Fig. 1*C*). The number of Antimycin A–damaged muscles also increased in a dose-dependent manner; ∼43 and 50% muscles are dystrophic after 2 and 4 µM Antimycin A treatment, which increases to ∼70% following 10 µM treatment. Notably, although we found a significant percentage of muscle damage following exposure to 2 µM Antimycin A, we did not observe paralysis in this condition, suggesting a certain degree of mitochondrial dysfunction–induced muscle damage can be tolerated without translating to a functional decline. At first glance, it can be predicted that paralysis is caused as a result of depletion of ATP, which is an established feature of Antimycin A treatment. Endogenous ATP levels after Antimycin A treatment distinctly reduced by more than half but did not change significantly between 2, 4, and 10 µM Antimycin A treatment compared with controls (Supplemental Fig. S1). Hence, ATP alone cannot explain the effect of Antimycin A treatment on the worm’s muscle cells.

### Mitochondrial dysfunction causes muscular ECM collagen degradation

**Figure 2:**
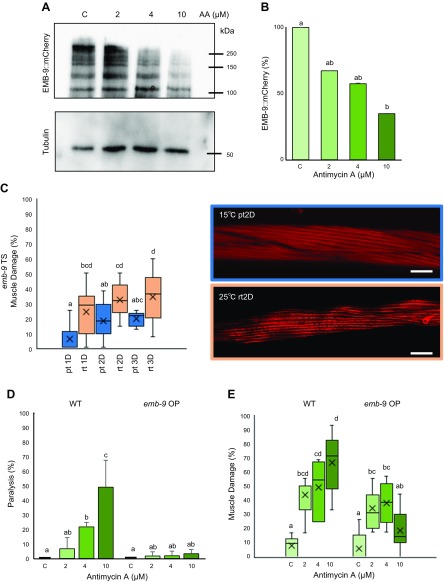
*emb-9* is degraded upon mitochondrial dysfunction caused by Antimycin A. *A*) Synchronized adult worms expressing emb-9::mCherry were treated with 0 (C, control), 2, 4, and 10 µM Antimycin A. After 36-h treatment with Antimycin A, animals were subjected to lysis, and Western blot was performed to analyze the amount of EMB-9 protein using anti-mCherry antibody. *B*) ImageJ was used to quantify the amount of EMB-9 protein, which was normalized to total tubulin content. Graph was prepared based on the values of the normalized amount of EMB-9; a significant decrease in EMB-9 was observed in 10 µM Antimycin A (3 biologic repeats). *C*) *emb-9* TS mutant animals were synchronized and grown to young adulthood at 15°C (permissive temperature), after which the adult worms were either grown at 15°C or shifted to 25°C for up to 3 d. At D1, D2 and D3, animals were monitored for muscle damage using rhodamine phalloidin staining and confocal microscopy. A significant increase in muscle damage was observed in animals grown at the restrictive temperature at D1, D2, and D3 when compared with their respective permissive temperature conditions. The pattern of this muscle damage is illustrated in the representative images. Number of muscle cells/condition (*n* = 260, 196, 270, 312, 177, and 347 from left to right). Scale bars, 10 µM. *D*, *E*) Antimycin A–treated *emb-9* OP worms were scored for worm paralysis and muscle damage as performed in Fig. 1*A, B*. Significant rescue of the paralysis phenotype was observed following 4 and 10 µM Antimycin A treatment, and muscle damage was recovered after 10 µM Antimycin A treatment. Twenty animals were analyzed for each experiment with 3 independent repetitions (*n* = 60 animals). Number of muscle cells/condition (*n* = 113, 106, 78, and 96 from left to right). The data for WT are the same as those used in Fig. 1*A, B*. They have been repeated here and in the following figures for comparison. OP, overexpression; pt, permissive temperature; rt, restrictive temperature. Letters (*a*–*d*) on top of bars indicate statistical significance; X, median value.

The ECM is crucial for not only maintaining the physical structure of a cell but also to provide it with an external biochemical environment that helps to mediate cellular activities *via* bilateral transmission of chemical cues. The predominant muscle dystrophy phenotype we observed was wavy actin filaments. Because of the wavy myofilament phenotype and previous reports highlighting the significance of muscle ECM (6), we considered whether a change in muscle attachment with the ECM or the composition of the ECM itself might be responsible for loss of myofilament anchoring and, therefore, wavy sarcomere structure. Collagen is the major ECM component, and we first analyzed changes in muscular collagen IV-α-1 (EMB-9 in *C. elegans*) after Antimycin A treatment using the NF3620 strain (*emb-9::mCherry*) (16). A progressive decrease in EMB-9::mCherry fusion protein was observed with increasing concentration of Antimycin A (**Fig. 2*A***), with 30 and 40% decreases in EMB-9 after 2 and 4 µM treatment. Significant decrease in EMB-9 was observed in the 10 µM Antimycin A condition with a degradation of ∼60% of EMB-9::mCherry fusion protein (Fig. 2*B*). We also observed a ∼30% decline in muscular LET-2 (collagen IV-α-2) protein after treatment with 4 and 10 µM Antimycin A through mass spectrometric analysis (Supplemental Table S1). To gain further evidence for the role of EMB-9 in muscle maintenance, we monitored *emb-9* mutant animals. Because EMB-9 is an essential protein, the absence of which leads to embryonic lethality, we employed TS *emb-9* mutants, which grow well at the permissive temperature (15°C) but fail to produce progeny at the restrictive temperature (25°C). We analyzed the muscle structure of adult *emb-9* mutants after 1, 2, and 3 d of incubation at the restrictive temperature and observed wavy actin filaments, which increase in a time-dependent manner (the percentage of dystrophic muscles increases from 25% on d 1 to 35% on d 3, Fig. 2*C*). However, it was also observed that the muscle damage seen in case of Antimycin A treatment, even at the 2 µM condition, was more severe than the one observed in the case of the *emb-9* mutant (Fig. 2*C*). This could be because more than 1 ECM protein is responsible for the muscle damage in case of Antimycin A treatment (*e.g.*, LET-2). It can be noted that muscle damage also increases at the permissive temperature in a time-dependent manner, which might be because of a leaky expression of the mutant protein. Furthermore, *emb-9* RNAi shows myofilament abnormalities in the muscle cells of MYO3::green fluorescent protein expressing *C. elegans* (Supplemental Fig. S2).

To determine whether EMB-9 positively regulates muscle structure and prevents muscle damage in the context of mitochondrial dysfunction, we examined the effect of EMB-9 overexpression in Antimycin A–treated NF3620 worms. We found that EMB-9 overexpression animals exhibit complete resistance to paralysis in all 3 concentrations of Antimycin A and, especially, there is significant rescue of paralysis in the 10 µM Antimycin A condition when compared with WT animals (Fig. 2*D*). We also found a decrease in muscle damage in the 2 and 4 µM Antimycin A treatment conditions, but a significant decrease was observed in the 10 µM Antimycin A condition compared with WT treatment conditions (Fig. 2*E*). These results clearly show the importance of muscular ECM collagens in maintaining and protecting muscle structure. Thus, our findings provide the first evidence that mitochondrial dysfunction serves as an intramuscular signal for ECM degradation and subsequent muscle damage.

### MMP activation mediates the ECM degradation

**Figure 3:**
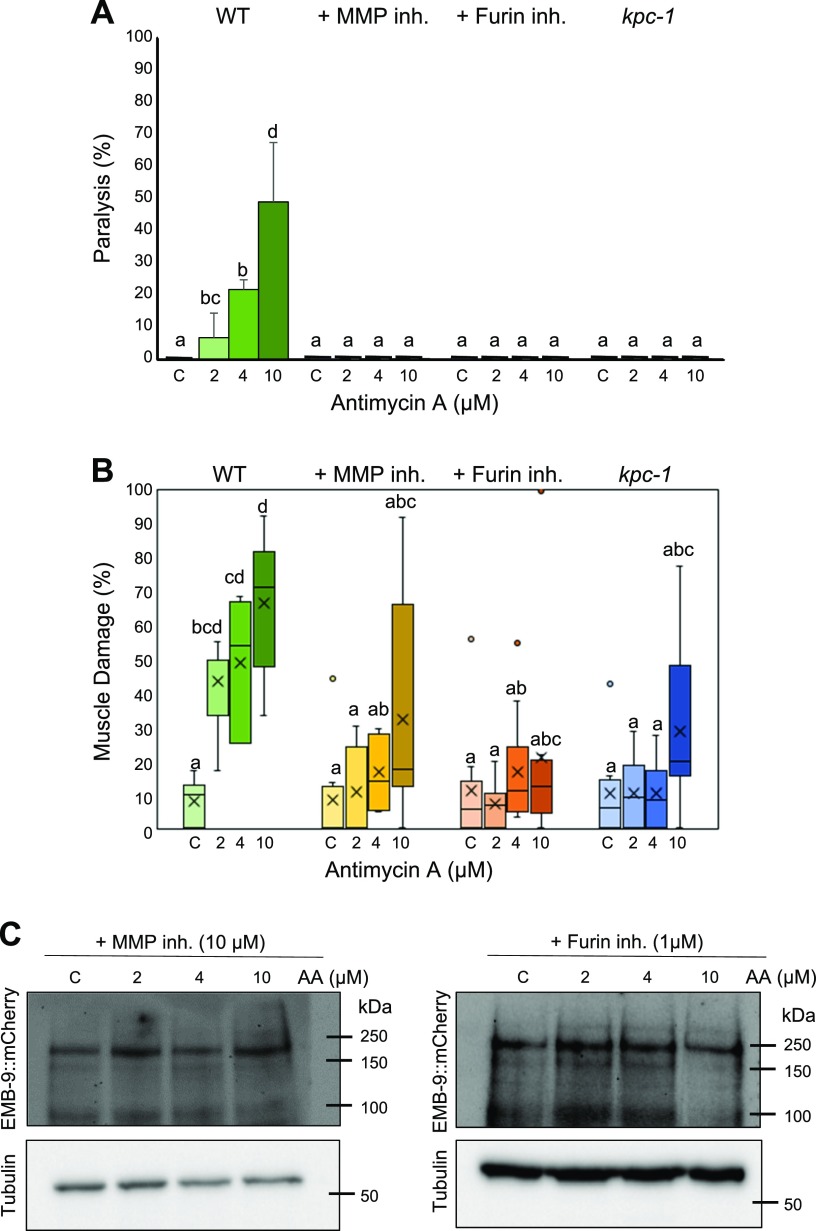
Inhibition of MMPs and Furin rescues the muscle paralysis, muscle damage, and collagen degradation mediated by Antimycin A. Adult synchronized animals (WT, *kpc-1*) were treated with 0 (C, control), 2, 4, and 10 µM Antimycin A along with 10 µM MMP inhibitor or 1 µM Furin inhibitor for 36 h, after which they were scored for worm paralysis and muscle damage. *A*) Significant rescue was observed in paralysis (2, 4, and 10 µM) in the presence of MMP inhibition or Furin inhibition. Twenty animals were analyzed for each experiment with 3 independent repetitions (*n* = 60 animals). *B*) Significant rescue was observed in muscle damage in 2, 4, and 10 µM Antimycin A treatment condition with MMP inhibitor, Furin inhibitor, and *kpc-1* mutant worms. Number of muscle cells/condition (*n* = 98, 116, 130, 102, 105, 126, 118, 165, 210, 342, 306, and 203 from left to right for MMP inhibitor, Furin inhibitor, and *kpc-1*). *C*) Synchronized adult worms expressing emb-9::mCherry were treated with 0, 2, 4, and 10 µM Antimycin A along with 10 µM MMP inhibitor or 1 µM Furin inhibitor. After 36-h treatment with Antimycin A, animals were subjected to lysis, and Western blot was performed to analyze the amount of EMB-9 protein using anti-mCherry antibody. No significant change in EMB-9 was observed with the application of MMP and Furin inhibitors (3 biologic repeats). Inh., inhibitor. Letters (*a*–*d*) on top of bars indicate statistical significance; X, median value.

MMPs are a set of proteases involved in the degradation of collagen and other ECM proteins. Furin is a proprotein convertase that is required for the activation of MMPs. Both Furin and MMPs require calcium for their activity (17–19). Because we noticed a decrease in EMB-9 and LET-2 protein after Antimycin A treatment, we next monitored the effect of MMP inhibition on Antimycin A–treated WT *C. elegans*. We found that MMP inhibition completely rescued worm paralysis in all 3 concentrations of Antimycin A (**Fig. 3*A***). Also, there was significant rescue of muscle damage in all 3 conditions when treated along with MMP inhibitor. The muscle damage dropped from ∼43, 50, and 70% in 2, 4, and 10 µM Antimycin A–treated animals to ∼10, 15, and 32% in animals treated with an MMP inhibitor along with Antimycin A (Fig. 3*B*). Additionally, both Furin mutant *kpc-1* and Furin inhibitor completely rescued paralysis caused by Antimycin A treatment (Fig. 3*A*). They also significantly rescued the number of damaged muscle cells from ∼43 and 50% to <15% in both 2 and 4 µM Antimycin A treatment conditions. It was interesting to observe that, even at the 10 µM Antimycin A condition, the muscle damage drastically reduced from 70 to 28% in the case of Furin mutant animals and 70 to 20% with the application of Furin inhibitor along with Antimycin A treatment (Fig. 3*B*). In addition to type-IV collagen (EMB-9 and LET-2), 2 cuticle collagens (COL-19 and COL-119) are also reduced by Antimycin A treatment (Supplemental Table S1). These are orthologs of human surfactant protein D, which is cleaved by MMP-9 *in vitro* (20). These results clearly indicate that mitochondrial dysfunction activates Furin and MMPs, which catalyze ECM collagen degradation. To confirm this, we analyzed whether MMP and Furin inhibitors could rescue EMB-9 degradation caused by Antimycin A treatment. We found no significant change in EMB-9 content with increasing concentrations of Antimycin A when treated along with MMP and Furin inhibitors (Fig. 3*C* and Supplemental Fig. S3*A*, *B*).

### Calcium signaling is up-regulated by mitochondrial dysfunction through the sarcoplasmic reticulum ryanodine receptor and the plasma membrane EGL-19 channel

**Figure 4:**
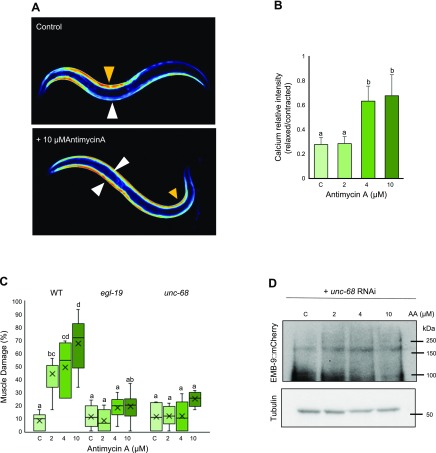
Inhibition of calcium overload could rescue muscle damage and EMB-9 degradation in Antimycin A–treated *C. elegans.* *A, B*) Animals expressing the calcium sensor GCaMP3.35 were age synchronized at the L1 stage and grown to young adulthood at 20°C. Adults were treated with 0 (C, control), 2, 4, and 10 µM Antimycin A for 36 h, after which they were imaged using fluorescence microscopy with objective lens, ×10 magnification (UPlanAPO, Olympus) (*A*) and the calcium level quantified (*B*) using ImageJ software. A significant rise in intracellular calcium was observed following treatment with 4 and 10 µM Antimycin A. Five animals were analyzed for each condition. *C*) Synchronized adult animals [WT, *unc-68*(*r1162*) V and *egl-19(ad695) IV*] were treated with 0 (C, control), 2, 4, and 10 µM Antimycin A for 36 h and scored for muscle damage as performed in Fig. 1*B*. When compared with WT animals, there was significant rescue or decrease in muscle damage in both mutants at 2, 4, and 10 µM Antimycin A. Number of muscle images/conditions (167, 218, 301, 273, 64, 80, 74, and 42 from left to right). *D*) Synchronized adult worms expressing emb-9::mCherry were treated with 0 (C, control), 2, 4, and 10 µM Antimycin A along with *unc-68* double-stranded RNA–containing bacteria. After 36-h treatment with Antimycin A, animals were subjected to lysis, and Western blot was performed to analyze the amount of EMB-9 protein using anti-mCherry antibody. No significant decrease in EMB-9 was observed with *unc-68* RNAi after Antimycin A treatment (3 biologic repeats). Letters (*a*–*d*) on top of bars indicate statistical significance; X, median value.

In one of our previous publications, we could see that heat stress leads to cytoplasmic calcium overload and mitochondrial fragmentation, possibly through endoplasmic reticulum (ER) stress. It has also been reported that reactive oxygen species (ROS) can also lead to calcium overload, and Antimycin A treatment is known to enhance both mitochondrial ROS as well as cytoplasmic ROS (21). Because calcium is required for the activation of both Furin and certain MMPs, we proceeded to examine the role of calcium in Antimycin A–mediated muscle damage. For this, we employed a muscular GCaMP calcium sensor, a genetically encoded calcium indicator (11, 22). We found rise in muscle cytoplasmic calcium levels with increasing concentrations of Antimycin A treatment, which correlates well with the progressive increase in muscle damage observed before. As the calcium current is essential for muscle contraction, a calcium rise can be seen in the contracted side (orange arrowhead) of the body in WT controls. However, in Antimycin A–treated animals, the calcium rise is visible throughout the body wall muscle regardless of muscle contraction, both on the contracted as well as the relaxed side (white arrowhead) (**Fig. 4*A***). When we calculated the ratio of the relative intensity of calcium on the relaxed side to the contracted side, we found a significant increase in 4 and 10 µM Antimycin A (Fig. 4*B*). Subsequently, we had to determine the mechanisms regulating this calcium overload caused by mitochondrial dysfunction. The ryanodine receptor (RyR) UNC-68 is one of the major intracellular calcium channels on the sarcoplasmic reticulum (SR) for calcium release (23), and EGL-19, a voltage-gated calcium channel, localizes on the muscle plasma membrane for entry of calcium from outside the cell (24). Null mutants of *egl-19* are lethal, whereas a reduction of function causes feeble contraction of body wall muscle cells, possibly because of reduced calcium entry (24, 25). RyRs, including UNC-68, also are responsible for excitation-contraction coupling through calcium induced calcium release from the SR (26). Mutation in *unc-68* as well as *egl-19* significantly rescued the muscle damage in all 3 concentrations of Antimycin A, decreasing it from 43–10% and 7% in 2 µM, 49–12% and 17% in 4 µM, and 70–25% and 18% in 10 µM Antimycin A in *unc-68* and *egl-19* mutants, respectively (Fig. 4*C*). Thus, the reduction of calcium entry into the cell through EGL-19 as well as reduced release of calcium from the SR into cytoplasm through UNC-68 could rescue the Antimycin A–induced muscle damage. Moreover, *unc-68* RNAi could also rescue EMB-9 degradation significantly, suggesting that calcium overload through these channels is responsible for the activation of Furin and MMPs (Fig. 4*D* and Supplemental Fig. S3*C*). The increase in calcium could be the direct result of ROS generated as a by-product of mitochondrial dysfunction because it has been shown that ROS-mediated oxidation of RyR (UNC-68) can increase its activity, resulting in cytosolic calcium overload (21).

### Rotenone-mediated muscle damage has a different mechanism than Antimycin A

**Figure 5:**
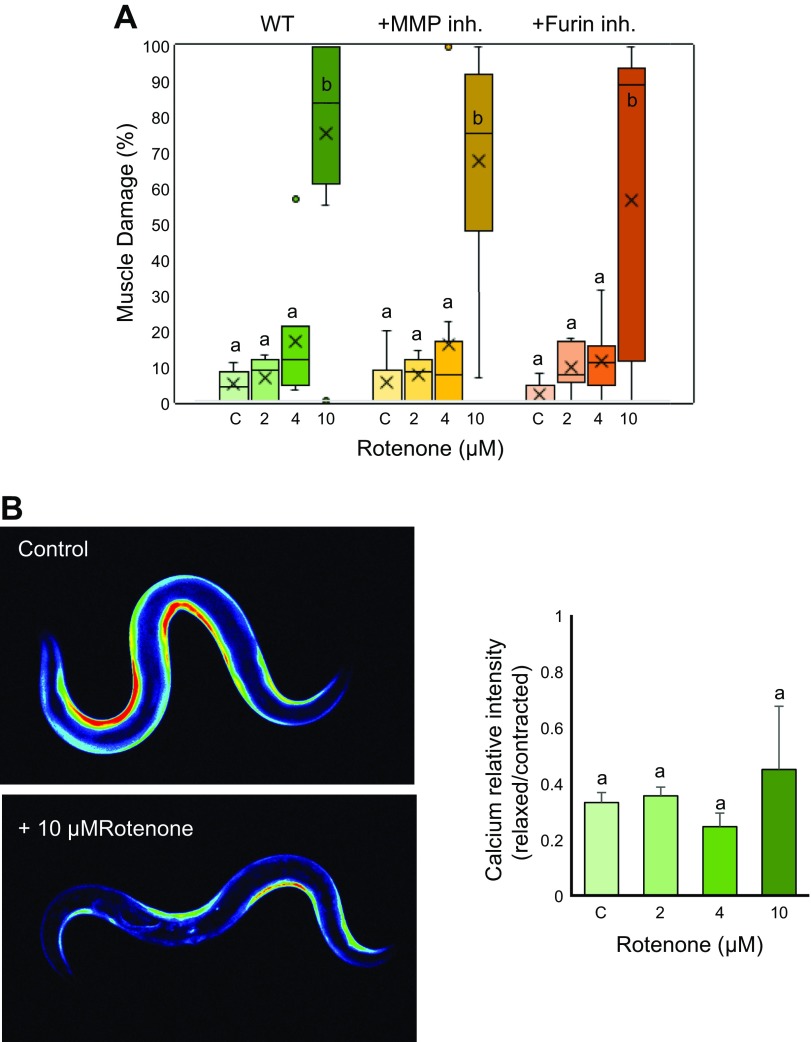
Rotenone treatment causes muscle damage but not calcium overload in *C. elegans.* *A*) Adult synchronized animals were treated with 0 (C, control), 2, 4 and 10 µM Rotenone for 36 h with or without MMP inhibitor (10 µM) or Furin inhibitor (1 µM), after which they were scored for muscle damage. Significant muscle damage was found in 10 µM rotenone, which could not be rescued with MMP or Furin inhibitor. Number of muscle images/conditions (269, 231, 144, 197, 280, 228, 207, 112, 257, 155, 217, and 169 from left to right). *B*) Adults expressing GCaMP3.35 were treated with 0 (C, control), 2, 4 and 10 µM Rotenone for 36 h, after which they were imaged using fluorescence microscopy with objective lens, ×10 magnification, and the calcium level quantified using ImageJ software. No significant change in calcium was observed in all 3 concentrations. Five animals were analyzed for each condition. Inh., inhibitor. Letters (*a, b*) on top of bars indicate statistical significance; X, median value.

Because we could successfully show that mitochondrial dysfunction mediated by Antimycin A causes muscle damage through ECM degradation, we were interested to see whether similar effects would appear with other mitochondrial inhibitors. We used rotenone in the same concentrations as Antimycin A because the OCR was reduced in a similar manner between both treatments (Supplemental Fig. S5*A*, *B*). Intriguingly, rotenone did not affect the muscle cells in a way similar to Antimycin A. We found no muscle damage with 2 and 4 µM rotenone treatment, but significant muscle damage was seen in the 10 µM condition (**Fig. 5*A*** and Supplemental Fig. S4). Because calcium overload is one of the earliest responses for the muscle damage in the Antimycin A model, we examined the calcium concentration in rotenone-treated worms. Surprisingly, we found no significant rise in calcium in all 3 concentrations of rotenone (Fig. 5*B*). We also examined whether the muscle damage observed in the 10 µM rotenone condition could be rescued with Furin or MMP inhibition. We found that both the inhibitors fail to rescue muscle damage observed in 10 µM rotenone–treated worms (Fig. 5*A*). Because calcium overload is essential for Furin and MMP activation, this result was expected. It can be concluded that cytoplasmic calcium overload is responsible for muscle damage in Antimycin A–treated worms, especially because rotenone treatment generates mitochondrial ROS, which is restricted inside the mitochondrial matrix and hence cannot oxidize RyR and cause cytoplasmic calcium overload (27). Thus, in the case of rotenone treatment, alternative pathways might be responsible for muscle damage at very high (10 µM) doses.

### Duchenne muscular dystrophy muscle decline is rescued by Furin and MMP inhibition

**Figure 6:**
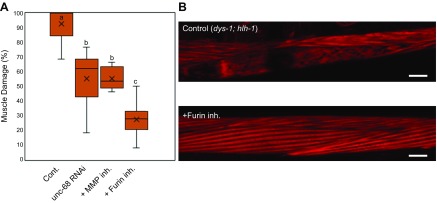
DMD muscle phenotype can be rescued with Furin inhibition. *A*) *dys-1; hlh-1* mutant animals were synchronized from the L1 stage and grown to young adulthood at 15°C (permissive temperature). Adult animals were then transferred to fresh NGM plates containing 1 µM Furin inhibitor or 10 µM MMP inhibitor or *unc-68* RNAi plates and grown at 25°C (restrictive temperature) for 48 h. There was a significant decrease in the occurrence of muscle damage in *dys-1*; *hlh-1* animals when treated with 1 µM Furin inhibitor, 10 µM MMP inhibitor, and *unc-68* RNAi. *B*) Representative image of a muscle cell of *dys-1*; *hlh-1* animals in the absence and presence of a Furin inhibitor. Number of muscle cell images/condition (*n* = 152, 109, 217, and 99 from left to right). Here, control animals are DMD animals treated with DMSO. Cont., control; inh., inhibitor. Scale bars, 10 µM. Letters (*a*–*c*) on top of bars indicate statistical significance; X, median value.

Duchenne muscular dystrophy (DMD) is a genetic disorder caused by a mutation in the dystrophin gene. Dystrophin is responsible for the maintenance of plasma membrane stability, and its absence leads to muscular dystrophy. Previous literature has reported calcium dysregulation and mitochondrial dysfunction in DMD, (28, 29) and, more recently, reduced mitochondrial function and calcium overload has been demonstrated in a *C. elegans* model of DMD (30, 31). Because the dystrophin gene is conserved in *C. elegans*, the well-established DMD model of *C. elegans* (*dys-1; hlh-1* mutant) is a good model system to further explore the role of mitochondrial dysfunction or ECM degradation in muscle maintenance (15). To validate our findings regarding the importance of collagen in protecting muscle cells against dystrophy, we examined whether Furin and MMP inhibition could suppress muscle damage in the DMD-model worms. The mutant accumulates a high degree of muscle damage (15), and we found 93% of muscle cells with damaged myofilaments that, although higher than that seen with Antimycin A treatment, displayed a similar pattern of wavy and broken myofilament (**Fig. 6*A, B***). Intriguingly, treatment with Furin or an MMP inhibitor resulted in a significant reduction in muscle damage, from 93 to 27% upon Furin inhibition and 55% with MMP inhibition (Fig. 6*A, B*). Likewise, *unc-68* inhibition through RNAi significantly rescued muscle damage in *dys-1*; *hlh-1* mutants from 93 to 55% (Fig. 6*A*). This leads us to conclude that calcium overload is the first of multiple steps by which chemical- (Antimycin A) and disease (DMD)-related mitochondrial dysfunction causes muscle damage through ECM degradation.

## DISCUSSION

In this study, we have shown ECM degradation as a novel pathway of muscle degeneration whereby reduced or altered collagen can result in muscle damage. The results corroborate previous studies conducted in collagen-null mice and cell lines that have provided important insight into the function of ECM with respect to mitochondrial function and muscle cell apoptosis as well as defective autophagy mechanism (7, 32). Importantly, we found that maintaining collagen content in the face of mitochondrial dysfunction through MMP and Furin inhibition could delay muscle damage for a significant period of time. Calcium overload was found to be one of the first steps in the sequential activation of ECM degradation, leading to muscle disorganization. Using forward genetics and mutant strains, we found that 2 important calcium receptors, *egl-19* and *unc-68*, are responsible for the calcium overload in *C. elegans* muscle. Mutation in either of these calcium receptors (*egl-19* and *unc-68*) prevents excess cytosolic calcium accumulation, which results in rescue of the muscle damage phenotype. These results highlight cytosolic calcium influx as a key intermediary step in the muscle damage caused by mitochondrial dysfunction. Moreover, rotenone-mediated muscle damage could not be rescued significantly with the application of Furin and MMP inhibitors because of the absence of calcium overload, which acts as a crucial step for Furin and MMP activation. This could be because rotenone treatment generates ROS that is restricted to mitochondrial matrix and hence cannot activate calcium overload by oxidation of RyR (21, 27).

Calcium dysregulation is also one of the preliminary steps in DMD. Previous studies have elucidated the mechanism of disease manifestation in DMD through plasma membrane instability and excessive calcium influx. It is unclear how this calcium overload results in muscle degeneration in DMD. We found that we could significantly delay muscle degeneration in the *C. elegans* model of DMD through *unc-68*, MMP, and Furin inhibition. Our results indicate that ECM degradation can be one of the pathways that is activated in response to calcium overload, resulting in muscle degeneration in DMD as well as muscle damage mediated by Antimycin A.

**Figure 7:**
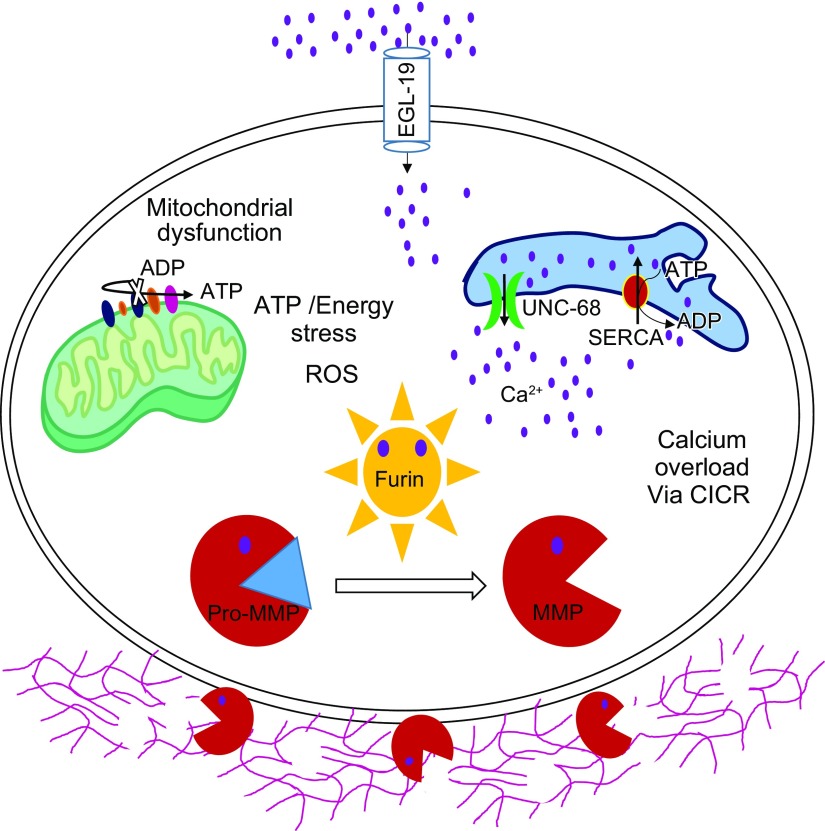
Collagen is essential for regulation of muscle cell activity and structure. In the case of mitochondrial dysfunction, as a first step, there is an up-regulation of intracellular calcium [possibly due to sarcoendoplasmic reticulum calcium transport ATPase (SERCA) loss of function and leaky RyR] that leads to Furin activation. Furin then further activates MMPs, which degrade collagen, an important ECM component. This then leads to muscle dystrophy. Preventing collagen degradation by inhibition of RyR, MMP, and Furin can delay muscle damage and keep the muscle structure intact for a longer time.

In summary, we propose a novel bilateral mechanism of muscle maintenance whereby mitochondrial dysfunction serves as an intramuscular cue for deregulated cytosolic calcium release, possibly through oxidation of UNC-68. This calcium overload subsequently activates the calcium-sensitive Furin-MMP-ECM degradation axis, which destabilizes sarcomeric myofilament anchoring to the ECM and ultimately leads to muscle structural decline and paralysis (**Fig. 7**). In addition, it can be speculated that ECM degradation also affects plasma membrane stability, causing further ionic influx. This would create a negative feedback loop ultimately resulting in muscle cell death through apoptosis. Dandrolene, a *unc-68* inhibitor, was found to be effective against DMD in the mouse model of DMD (33). Because MMP and Furin inhibition could attenuate mitochondrial dysfunction–mediated muscle damage, we propose MMP and Furin inhibitors as promising candidates for drugs against muscle-wasting conditions characterized by mitochondrial dysfunction, including DMD but also aging and disuse or bedrest.

## Supplementary Material

This article includes supplemental data. Please visit *http://www.fasebj.org* to obtain this information.

## Abbreviations

DMD: Duchenne muscular dystrophy
ECM: extracellular matrix
MMP: matrix metalloproteinase
NGM: Nematode growth medium
OCR: oxygen-consumption rate
RNAi: RNA interference
ROS: reactive oxygen species
RyR: ryanodine receptor
SR: sarcoplasmic reticulum
TS: temperature sensitive
WT: wild type
