# Supplementary material for: Mitochondrial dysfunction causes Ca2+ overload and ECM degradation–mediated muscle damage in C. elegans
Source: FASEB J. 2019 Jun 4;33(8):9540–50. doi: 10.1096/fj.201802298R (PMC6662967; doi:10.1096/fj.201802298R)
Supplement: Supplementary file 1 [file fj.201802298R.sd1.pdf]

Supplementary legends:

Supplementary Figure 1: ATP reduction was observed in *C. elegans* after Antimycin A treatment. Age synchronized wild-type animals were grown to young adulthood at 20°C. Adult animals were then treated with 0, 2, 4 and 10 μM Antimycin A for 24 hours. An ATP analysis kit was used to measure the endogenous level in the worms after treatment with Antimycin A.

Supplementary Figure 2: *emb-9* depletion causes muscle defects. *myo-3p*::Myo3::GFP expressing adult worms were transferred to *emb-9* RNAi plates for 48 hours, after which they were examined for muscle damage. Muscle structure was seen to be distorted after *emb-9* RNAi treatment. Scale bar: 10 μM.

Supplementary Figure 3: MMP, Furin, and UNC-68 inhibition could rescue collagen degradation caused by Antimycin A. Synchronized adult worms expressing *emb-9::mCherry* were treated with 0, 2, 4 and 10 μM Antimycin A along with (A) 10 μM MMP inhibitor or (B) 1 μM Furin inhibitor or (C) *unc-68* RNAi. After 36 hour treatment with Antimycin A, animals were subjected to lysis and western blot was performed to analyze the amount of EMB-9 protein using anti-mCherry antibody. Image J was used to quantify the amount of EMB-9 protein, which was normalized to total tubulin content. The graph represents the values of normalized amount of EMB-9; no significant decrease in EMB-9 was observed with the application of MMP and Furin inhibitors or *unc-68* RNAi. (3 biological repeats)

Supplementary Figure 4: 10 μM Rotenone treatment causes muscle damage. Image

representing normal muscle in case of control (1% DMSO) and damaged muscle in case of 10 μM Rotenone treatment has been shown. Scale bar: 10 μM.

Supplementary Figure 5: Antimycin A and Rotenone treatments dose-dependently disrupt mitochondrial respiratory function. Treatment of adult *C. elegans* for 36 hours with 2, 4 and 10 μM Antimycin A or Rotenone significantly reduces oxygen consumption rate (OCR) in a dose dependent manner. Data is the composite of 2 separate experiments, totaling 10 technical replicates and 200 animals per condition. * denotes $P < 0.05$, **** denotes $P < 0.0001$.

Supplementary Table 1: EMB-9 homologue LET-2, COL-19, and COL-119 were downregulated after Antimycin A treatment. Adult synchronized *C. elegans*, treated with 4 μM and 10 μM Antimycin A for 24 hours, were subjected to trypsin digestion and peptide labelling using an iTRAQ labeling kit (Applied Biosystems). Mass spectrometry was performed using an LTQ Orbitrap Velos with ETD mass spectrometer (Thermo Fisher Scientific).
