# Supplementary material for: Mitochondrial dysfunction causes Ca2+ overload and ECM degradation–mediated muscle damage in C. elegans
Source: FASEB J. 2019 Jun 4;33(8):9540–50. doi: 10.1096/fj.201802298R (PMC6662967; doi:10.1096/fj.201802298R)
Supplement: Supplementary file 2 [file fj.201802298R.sd2.pptx]

## Slide 1
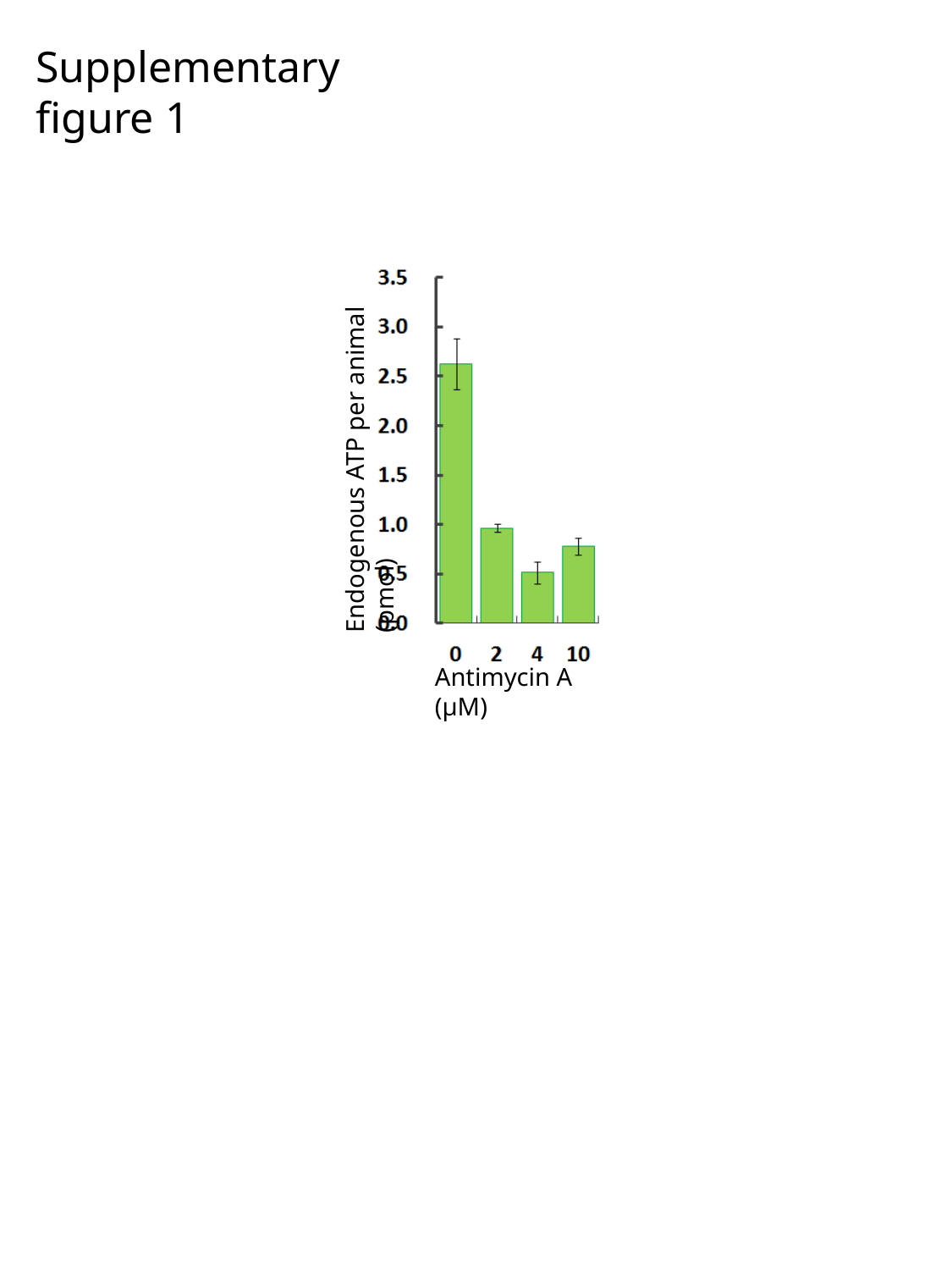

Supplementary figure 1
Endogenous ATP per animal (pmol)
Antimycin A (µM)

## Slide 2
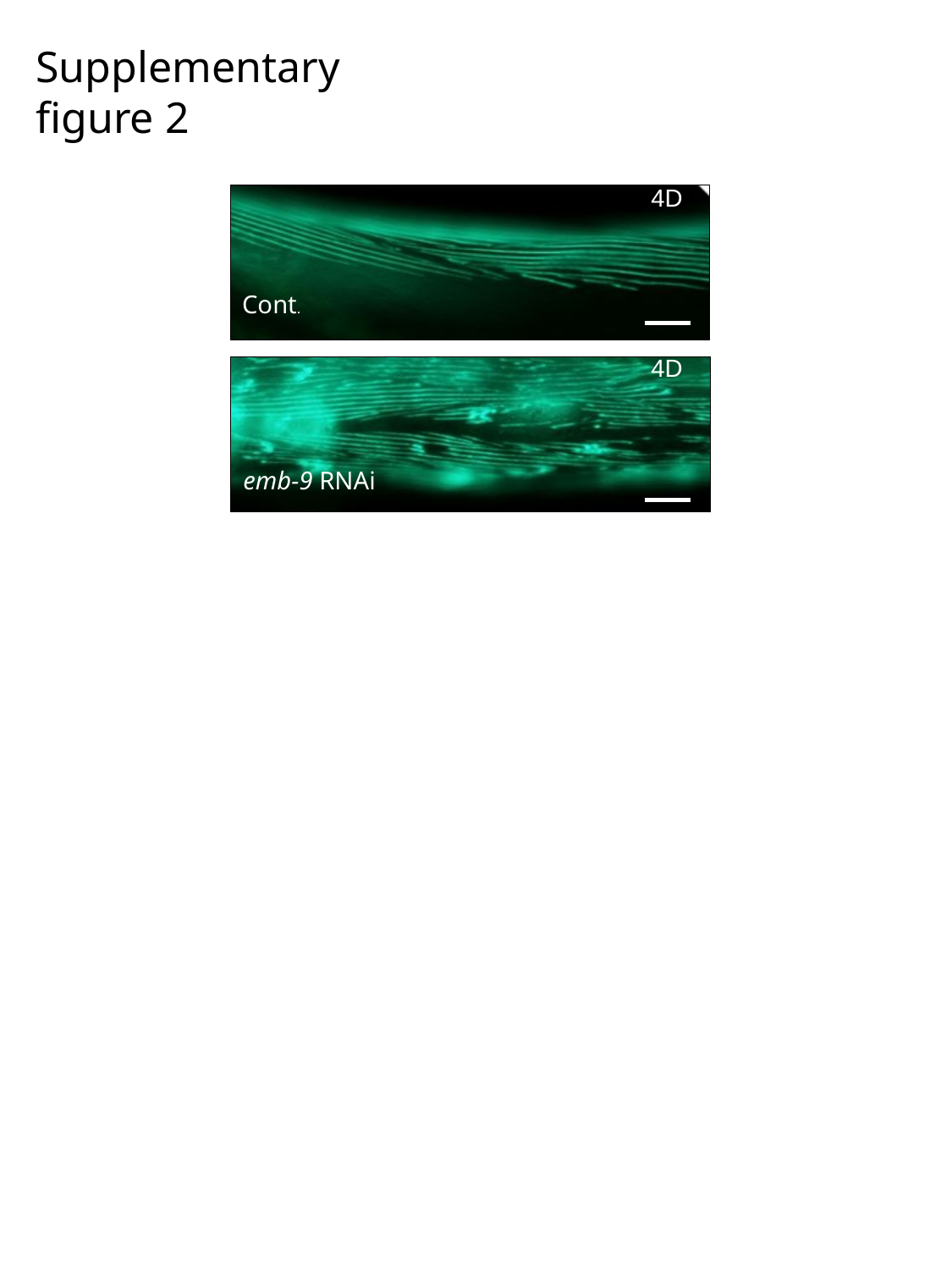

Supplementary figure 2
4D
Cont.
4D
emb-9 RNAi

## Slide 3
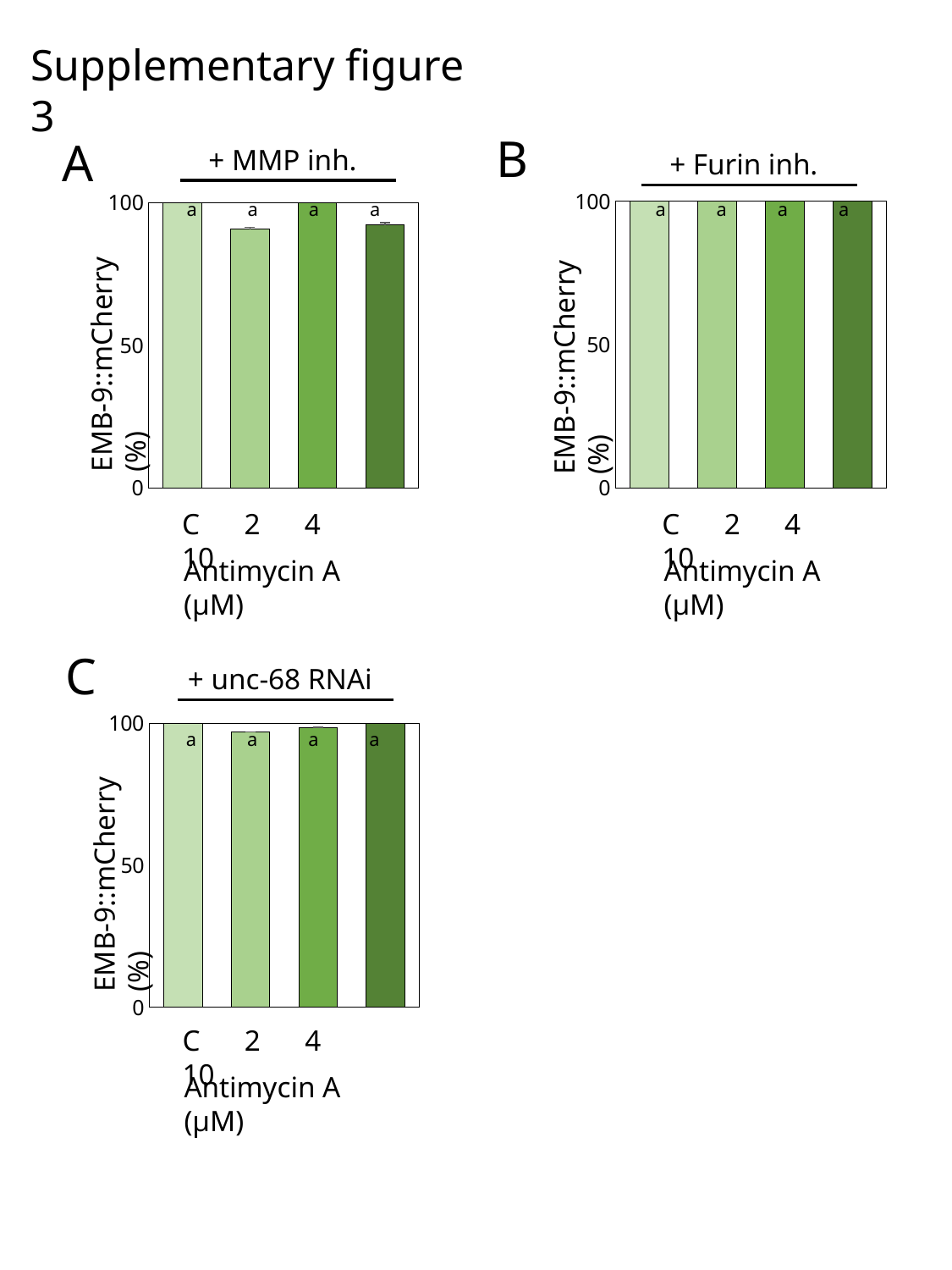

Supplementary figure 3
B
A
+ MMP inh.
+ Furin inh.
### Chart
| Category | |
|---|---|
| Control | 100.0 |
| 2 uM AA | 102.49 |
| 4 uM AA | 110.43 |
| 10 uM AA | 116.46 |
### Chart
| Category | |
|---|---|
| Control | 100.0 |
| 2uMAA | 90.75 |
| 4uMAA | 102.98 |
| 10uMAA | 92.26 |a
a
a
a
a
a
a
a
EMB-9::mCherry (%)
EMB-9::mCherry (%)
C 2 4 10
C 2 4 10
Antimycin A (µM)
Antimycin A (µM)
C
+ unc-68 RNAi
### Chart
| Category | |
|---|---|
| Control | 100.0 |
| 2uM AA | 96.84 |
| 4uM AA | 98.41 |
| 10uM AA | 106.28 |a
a
a
a
EMB-9::mCherry (%)
C 2 4 10
Antimycin A (µM)

## Slide 4
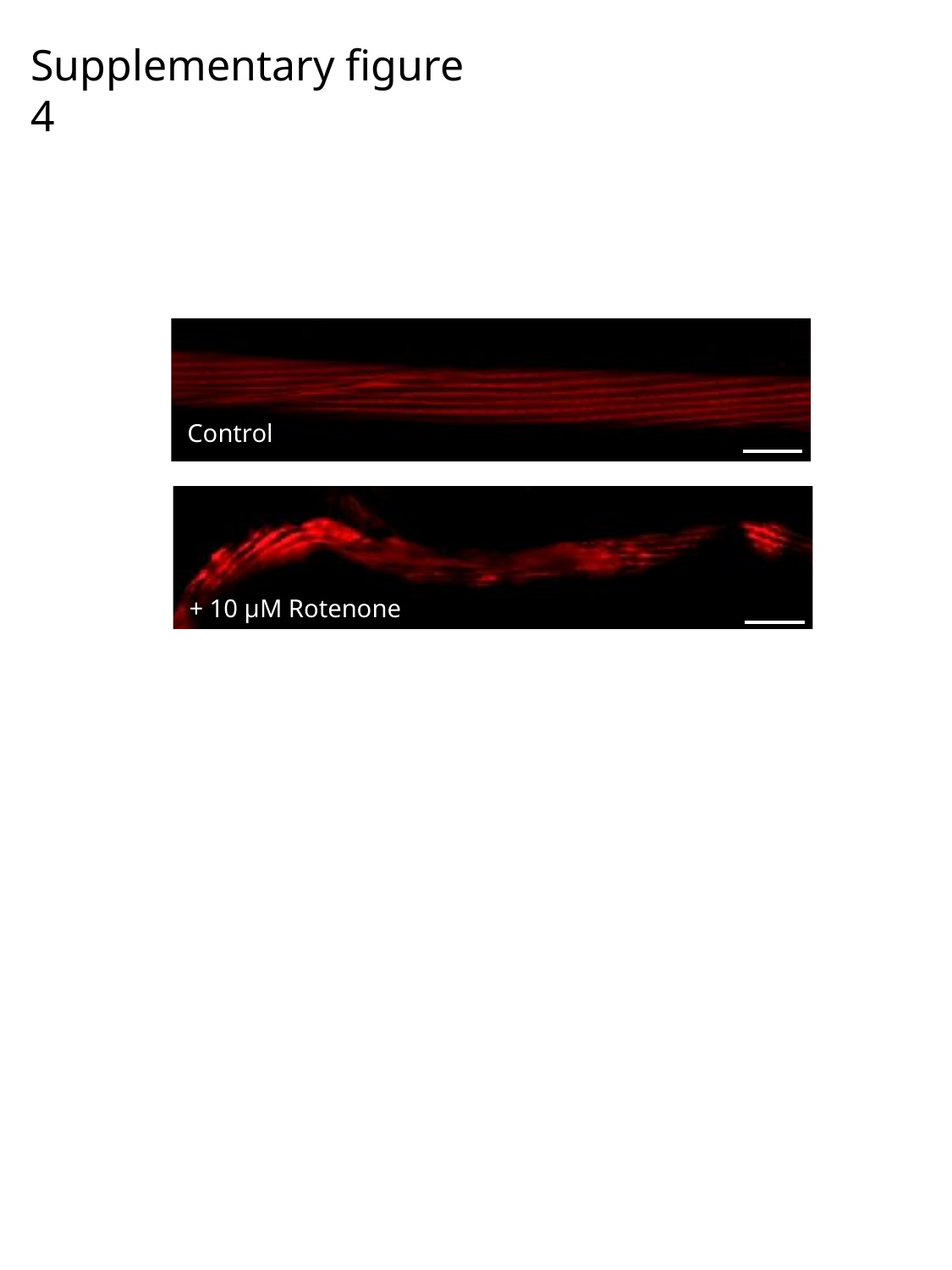

Supplementary figure 4
Control
+ 10 µM Rotenone

## Slide 5
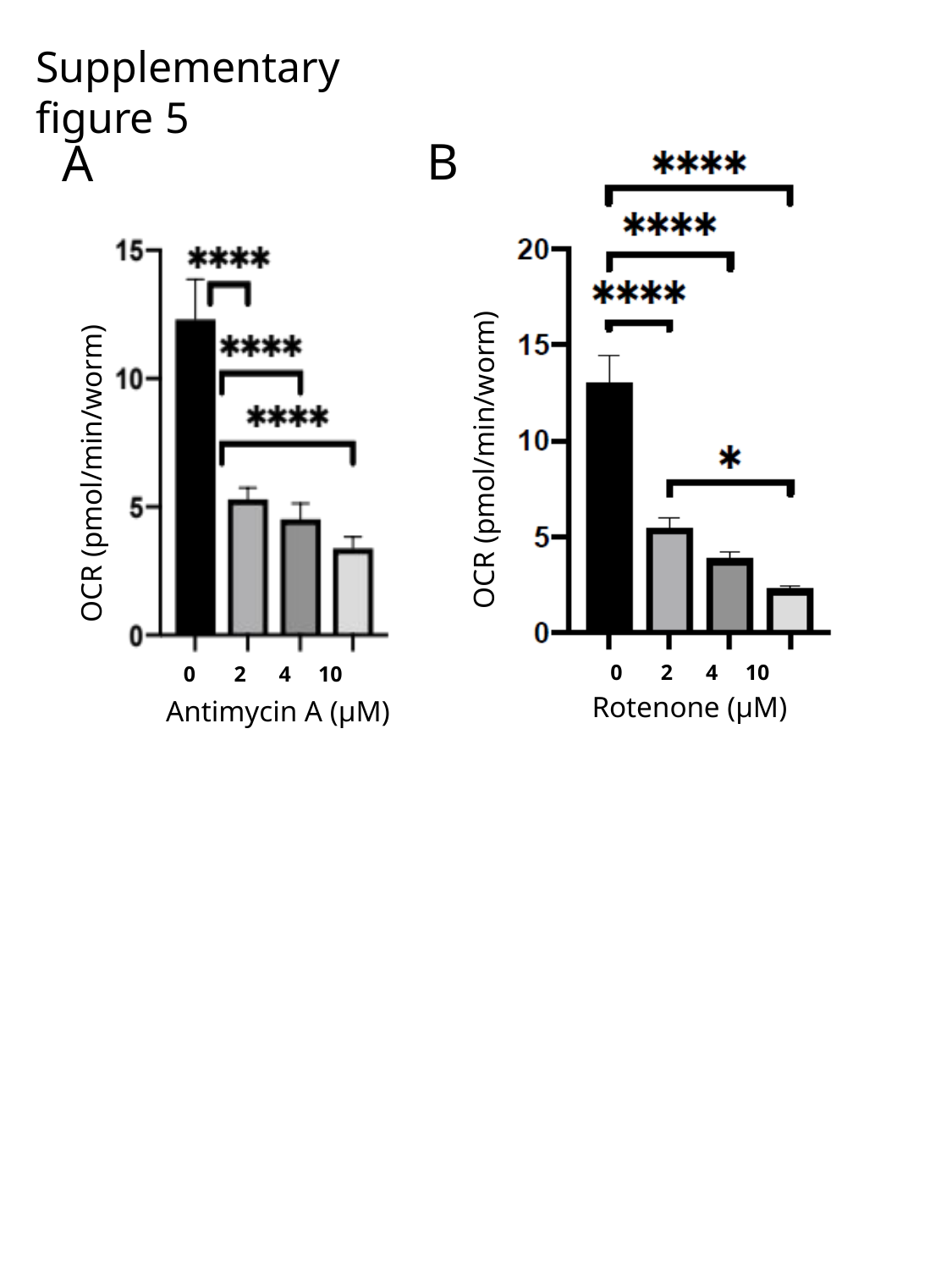

Supplementary figure 5
B
A
OCR (pmol/min/worm)
0 2 4 10
Rotenone (µM)
OCR (pmol/min/worm)
0 2 4 10
Antimycin A (µM)

## Slide 6
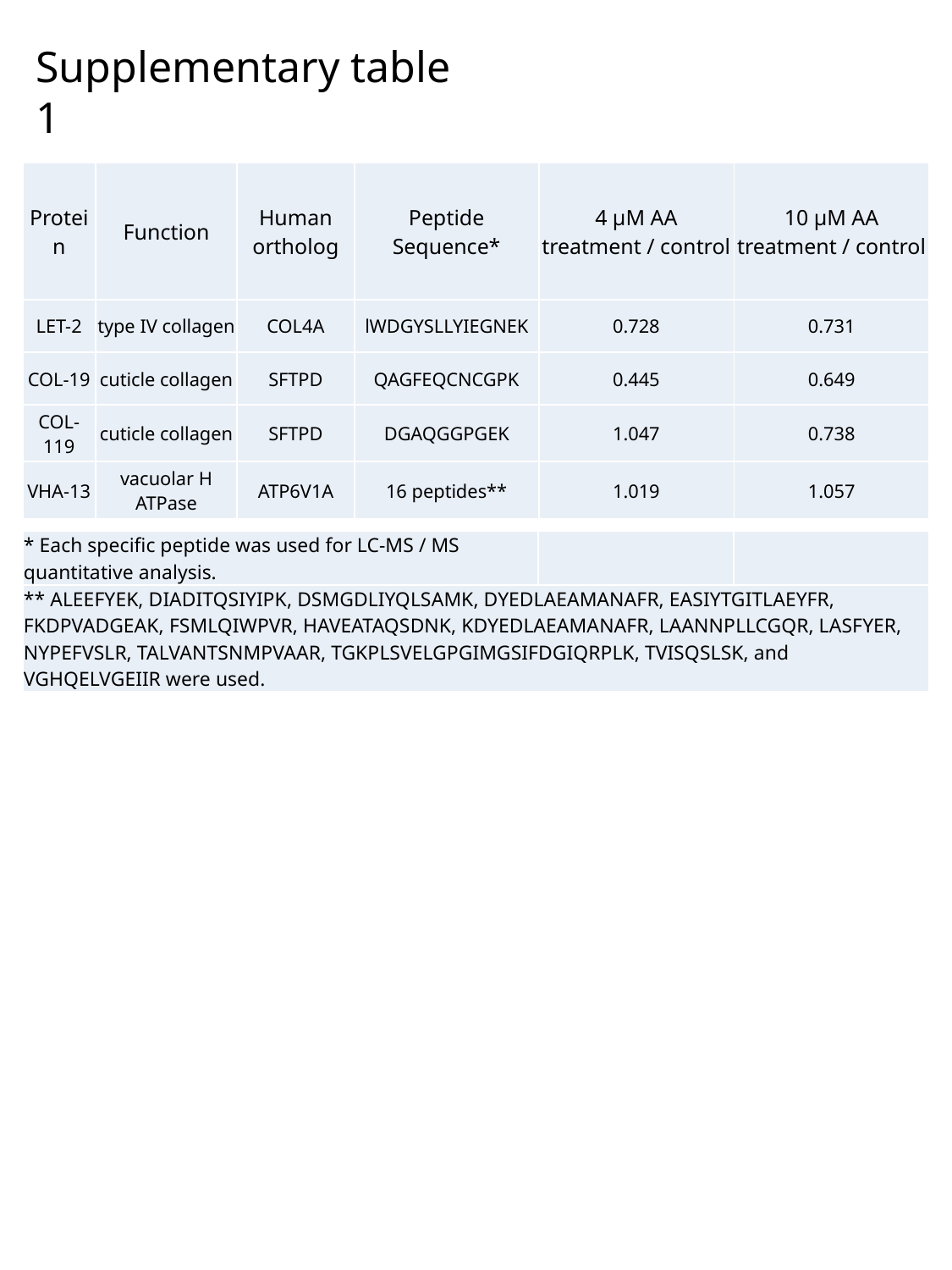

Supplementary table 1
| Protein | Function | Human ortholog | Peptide Sequence\* | 4 µM AA treatment / control | 10 µM AA treatment / control |
| --- | --- | --- | --- | --- | --- |
| LET-2 | type IV collagen | COL4A | lWDGYSLLYIEGNEK | 0.728 | 0.731 |
| COL-19 | cuticle collagen | SFTPD | QAGFEQCNCGPK | 0.445 | 0.649 |
| COL-119 | cuticle collagen | SFTPD | DGAQGGPGEK | 1.047 | 0.738 |
| VHA-13 | vacuolar H ATPase | ATP6V1A | 16 peptides\*\* | 1.019 | 1.057 |
| \* Each specific peptide was used for LC-MS / MS quantitative analysis. | | |
| --- | --- | --- |
| \*\* ALEEFYEK, DIADITQSIYIPK, DSMGDLIYQLSAMK, DYEDLAEAMANAFR, EASIYTGITLAEYFR, FKDPVADGEAK, FSMLQIWPVR, HAVEATAQSDNK, KDYEDLAEAMANAFR, LAANNPLLCGQR, LASFYER, NYPEFVSLR, TALVANTSNMPVAAR, TGKPLSVELGPGIMGSIFDGIQRPLK, TVISQSLSK, and VGHQELVGEIIR were used. | | |
